# Supplementary material for: Occurrence and Characteristics of Serpula himantioides Fruiting Bodies on Living Trees in Japan
Source: J Fungi (Basel). 2024 Aug 14;10(8):572. doi: 10.3390/jof10080572 (PMC11355443; doi:10.3390/jof10080572)
Supplement: Supplementary file 1 [file jof-10-00572-s001.zip › Jof_SupplementaryV3.pdf]

Table S1. Locations and main characteristics of the study plots

**Plot Name**	**A**	**B**	**C**
Coordinates	N 35°56' 51"<br>E 138°49'22"	N 35°56' 32"<br>E 138°48' 26"	N 35°57' 09"<br>E 138°50' 49"
Elevation (m a.s.l.)	1153	1229	1084
Slope (°)	29	29	29
Aspect	SSE	SE	NNE
Planted tree species	*Chamaecyparis pisifera*	*Larix kaempferi*<br>*C. obtusa*	*Larix kaempferi*<br>*C. obtusa*
Stand age at 2023	94	86	86

Table S2. List of trees with *Serpula himantioides* fruiting bodies.

Tree number	Locality of source	Host	Collected Year	Bottom edge of fruiting body	Isolate number	Source of isolation	INSD[1] accession number: ITS
A1	Plot A	*Chamaecyparis pisifera*	2021	Contact with the ground	UTCF3112J1	Basidiospores	LC804819
A2	Plot A	*C. pisifera*	2021	Contact with the ground	UTCF3112J2	Basidiospores	LC804820
A3	Plot A	*C. pisifera*	2021	Contact with the ground	UTCF3112J3	Basidiospores	LC804821
A4	Plot A	*C. pisifera*	2021	Contact with the ground	UTCF3112J4	Basidiospores	LC804822
A5	Plot A	*C. pisifera*	2021	Contact with the ground	UTCF3112J5	Basidiospores	LC804823
A6	Plot A	*C. pisifera*	2021	Contact with the ground	UTCF3112J6	Basidiospores	LC804824
A7	Plot A	*C. pisifera*	2021	Contact with the ground	UTCF3112J7	Basidiospores	LC804825
A8	Plot A	*C. pisifera*	2021	Contact with the ground	UTCF3112J8	Basidiospores	LC804826
A9	Plot A	*C. pisifera*	2021	Contact with the ground	UTCF3112J9	Basidiospores	LC804827
A10	Plot A	*C. pisifera*	2021	Contact with the ground	UTCF3112J10	Basidiospores	LC804828
A11	Plot A	*C. pisifera*	2021	Contact with the ground	UTCF3112J11	Basidiospores	LC804829
A12	Plot A	*C. pisifera*	2022	Contact with the ground	UTCF3112J22	Basidiospores	LC804840
A13	Plot A	*C. pisifera*	2022	Contact with the ground		Could not isolate	
A14	Plot A	*C. pisifera*	2022	Contact with the ground	UTCF3112J24	Basidiospores	LC804841
A15	Plot A	*C. pisifera*	2022	Contact with the ground		Could not isolate	

Results of PCR using species-specific primer set of *S. himantioides* were all positive.

1: International Nucleotide Sequence Database

Table S2. List of trees with *Serpula himantioides* fruiting bodies (continuation).

Tree number	Locality of source	Host	Collected Year	Bottom edge of fruiting body	Isolate number	Source of isolation	INSD accession number: ITS
A16	Plot A	*Chamaecyparis pisifera*	2022	Contact with the ground		Could not isolate	
A17	Plot A	*C. pisifera*	2023	Contact with the ground		Could not isolate	
A18	Plot A	*C. pisifera*	2023	Contact with the ground		Could not isolate	
A19	Plot A	*C. pisifera*	2023	Contact with the ground		Could not isolate	
A20	Plot A	*C. pisifera*	2023	Contact with the ground		Could not isolate	
A21	Plot A	*C. pisifera*	2023	Contact with the ground	UTCF3112J32	Basidiospores	LC804842
A22	Plot A	*C. pisifera* (Dead Tree)	2023	Contact with the ground		Could not isolate	
B1	Plot B	*C. obtusa*	2021	Contact with the ground	UTCF2805L1	Basidiospores	LC804844
B2	Plot B	*Larix kaempferi*	2021	Contact with the ground	UTCF2805L2	Basidiospores	LC804845
B3	Plot B	*C. obtusa*	2021	Contact with the ground	UTCF2805L3	Basidiospores	LC804846
B4	Plot B	*C. obtusa*	2021	Contact with the ground	UTCF2805L4	Basidiospores	LC804847
B5	Plot B	*C. obtusa*	2021	Contact with the ground	UTCF2805L5	Basidiospores	LC804848
B6	Plot B	*C. obtusa*	2021	Contact with the ground	UTCF2805L6	Basidiospores	LC804849
B7	Plot B	*C. obtusa*	2021	Contact with the ground	UTCF2805L7	Basidiospores	LC804850
B8	Plot B	*C. obtusa*	2021	Contact with the ground	UTCF2805L8	Basidiospores	LC804851

Results of PCR using species-specific primer set of *S. himantioides* were all positive.

Table S2. List of trees with *Serpula himantioides* fruiting bodies (continuation).

Tree number	Locality of source	Host	Collected Year	Bottom edge of fruiting body	Isolate number	Source of isolation	INSD accession number: ITS
B9	Plot B	*Chamaecyparis obtusa*	2021	Contact with the ground	UTCF2805L9	Basidiospores	LC804852
B10	Plot B	*C. obtusa*	2021	Contact with the ground	UTCF2805L10	Basidiospores	LC804853
B11	Plot B	*C. obtusa*	2021	Contact with the ground	UTCF2805L11	Basidiospores	LC804854
B12	Plot B	*Larix kaempferi*	2021	Contact with the ground	UTCF2805L12	Basidiospores	LC804855
B13	Plot B	*C. obtusa*	2021	Contact with the ground	UTCF2805L13	Basidiospores	LC804856
B14	Plot B	*L. kaempferi*	2021	Contact with the ground	UTCF2805L14	Basidiospores	LC804857
B15	Plot B	*L. kaempferi*	2021	Contact with the ground	UTCF2805L15	Basidiospores	LC804858
B16	Plot B	*L. kaempferi*	2021	Contact with the ground	UTCF2805L16	Basidiospores	LC804859
B17	Plot B	*C. obtusa*	2021	Contact with the ground	UTCF2805L17	Basidiospores	LC804860
B18	Plot B	*L. kaempferi*	2022	Contact with the ground	UTCF2805L18	Basidiospores	LC804861
B19	Plot B	*L. kaempferi*	2022	Contact with the ground	UTCF2805L19	Basidiospores	LC804862
B20	Plot B	*L. kaempferi*	2022	Contact with the ground		Could not isolate	
B21	Plot B	*C. obtusa*	2023	Contact with the ground	UTCF2805L21	Basidiospores	LC804863
B22	Plot B	*L. kaempferi*	2023	Contact with the ground		Basidiospores	
B23	Plot B	*L. kaempferi*	2023	Contact with the ground		Could not isolate	

Results of PCR using species-specific primer set of *S. himantioides* were all positive.

Table S2. List of trees with *Serpula himantioides* fruiting bodies (continuation).

Tree number	Locality of source	Host	Collected Year	Bottom edge of fruiting body	Isolate number	Source of isolation	INSD accession number: ITS
B24	Plot B	*Larix kaempferi*	2023	Contact with the ground		Could not isolate	
C1	Plot C	*Chamaecyparis obtusa*	2021	Contact with the ground	UTCF3204K1	Basidiospores	LC804864
C2	Plot C	*L. kaempferi*	2021	Contact with the ground	UTCF3204K2	Basidiospores	LC804865
C3	Plot C	*C. obtusa*	2021	Contact with the ground	UTCF3204K3	Basidiospores	LC804866
C4	Plot C	*L. kaempferi*	2021	Contact with the ground	UTCF3204K4	Basidiospores	LC804867
C5	Plot C	*C. obtusa*	2021	Contact with the ground	UTCF3204K5	Basidiospores	LC804868
C6	Plot C	*C. obtusa*	2021	Contact with the ground	UTCF3204K6	Basidiospores	LC804869
C7	Plot C	*C. obtusa*	2021	Contact with the ground	UTCF3204K7	Basidiospores	LC804870
C8	Plot C	*C. obtusa*	2021	Contact with the ground	UTCF3204K8	Basidiospores	LC804871
C9	Plot C	*C. obtusa*	2021	Contact with the ground	UTCF3204K9	Basidiospores	LC804872
C10	Plot C	*L. kaempferi*	2021	Contact with the ground	UTCF3204K10	Basidiospores	LC804873
C11	Plot C	*L. kaempferi*	2021	Contact with the ground	UTCF3204K11	Could not isolate	LC804874
C12	Plot C	*L. kaempferi*	2021	Contact with the ground	UTCF3204K12	Basidiospores	LC804875
C13	Plot C	*C. obtusa*	2021	Contact with the ground	UTCF3204K13	Basidiospores	LC804876
C14	Plot C	*C. obtusa*	2021	Contact with the ground	UTCF3204K14	Basidiospores	LC804877

Results of PCR using species-specific primer set of *S. himantioides* were all positive.

Table S2. List of trees with *Serpula himantioides* fruiting bodies (continuation).

Tree number	Locality of source	Host	Collected Year	Bottom edge of fruiting body	Isolate number	Source of isolation	INSD accession number: ITS
C15	Plot C	*Chamaecyparis obtusa* (Dead tree)	2021	Contact with the ground	UTCF3204K15	Basidiospores	LC804878
C16	Plot C	*C. obtusa*	2021	Contact with the ground	UTCF3204K16	Basidiospores	LC804879
C17	Plot C	*C. obtusa*	2021	Contact with the ground	UTCF3204K17	Basidiospores	LC804880
C18	Plot C	*Larix kaempferi*	2021	Contact with the ground	UTCF3204K18	Basidiospores	LC804881
C19	Plot C	*C. obtusa*	2021	Contact with the ground	UTCF3204K19	Basidiospores	LC804882
C20	Plot C	*C. obtusa*	2021	Contact with the ground	UTCF3204K20	Basidiospores	LC804883
C21	Plot C	*L. kaempferi*	2021	Contact with the ground	UTCF3204K21	Basidiospores	LC804884
C22	Plot C	*C. obtusa*	2021	Contact with the ground	UTCF3204K22	Basidiospores	LC804885
C23	Plot C	*L. kaempferi*	2021	Contact with the ground	UTCF3204K23	Basidiospores	LC804886
C24	Plot C	*L. kaempferi*	2021	Contact with the ground	UTCF3204K24	Basidiospores	LC804887
C25	Plot C	*C. obtusa*	2021	Contact with the ground	UTCF3204K25	Basidiospores	LC804888
C26	Plot C	*L. kaempferi* (Dead tree)	2021	Contact with the ground	UTCF3204K26	Could not isolate	LC804889
C27	Plot C	*C. obtusa*	2022	Contact with the ground	UTCF3204K27	Basidiospores	LC804890
C28	Plot C	*L. kaempferi*	2022	Contact with the ground	UTCF3204K28	Basidiospores	LC804891
C29	Plot C	*C. obtusa*	2022	Contact with the ground		Could not isolate	

Results of PCR using species-specific primer set of *S. himantioides* were all positive.

Table S2. List of trees with *Serpula himantioides* fruiting bodies (continuation).

Tree number	Locality of source	Host	Collected Year	Bottom edge of fruiting body	Isolate number	Source of isolation	INSD accession number: ITS
C30	Plot C	*Chamaecyparis obtusa*	2022	Contact with the ground		Could not isolate	
C31	Plot C	*C. obtusa*	2022	Contact with the ground		Could not isolate	
C32	Plot C	*C. obtusa*	2023	Contact with the ground		Could not isolate	
C33	Plot C	*Larix kaempferi*	2023	Contact with the ground		Could not isolate	
C34	Plot C	*C. obtusa*	2023	Contact with the ground		Could not isolate	
C35	Plot C	*L. kaempferi*	2023	Contact with the ground		Could not isolate	
C36	Plot C	*C. obtusa*	2023	Contact with the ground		Could not isolate	
C37	Plot C	*L. kaempferi*	2023	Contact with the ground		Could not isolate	
C38	Plot C	*C. obtusa* (Dead tree)	2023	Contact with the ground		Could not isolate	
D1	Forest road	*C. pisifera*	2021	Contact with the ground	UTCF3114M1	Basidiospores	LC804892
D2	Forest road	*C. pisifera*	2021	Contact with the ground	UTCF3114M2	Basidiospores	LC804893
D3	Forest road	*C. pisifera*	2021	Contact with the ground	UTCF3114M3	Could not isolate	LC804894
D4	Plantation	*Cryptomeria japonica*	2021	Contact with the ground	UTCF1204M4	Basidiospores	LC804895
D5	Plantation	*C. japonica*	2021	Contact with the ground	UTCF20M5	Basidiospores	LC804896
D6	Plantation	Broadleaved tree (Dead tree)	2021	Contact with the ground		Could not isolate	

Results of PCR using species-specific primer set of *S. himantioides* were all positive.

Table S2. List of trees with *Serpula himantioides* fruiting bodies (continuation).

Tree number	Locality of source	Host	Collected Year	Bottom edge of fruiting body	Isolate number	Source of isolation	INSD accession number: ITS
D7	Plantation	*Clethra barbinervis* （Dead tree）	2021	Contact with the ground	UTCF3112M7	Basidiospores	LC804897
D8	Plantation	*Cryptomeria japonica*	2021	Contact with the ground	UTCF1204M8	Basidiospores	LC804898
D9	Plantation	*Chamaecyparis obtusa*	2022	No contact with the ground	UTCF0329M9	Basidiospores	LC804899
D10	Plantation	Broadleaved tree (Dead tree）	2022	No contact with the ground	UTCF0329M10	Basidiospores	LC804900
D11	Plantation	*C. japonica*	2022	Contact with the ground	UTCF2901N1	Basidiospores	LC804901
D12	Plantation	*C. japonica*	2022	Contact with the ground	UTCF2901N2	Basidiospores	LC804902
D13	Plantation	*C. japonica*	2022	Contact with the ground	UTCF2901N3	Basidiospores	LC804903
D14	Plantation	*C. japonica*	2022	Contact with the ground	UTCF2901N4	Basidiospores	LC804904
D15	Plantation	*C. japonica*	2022	Contact with the ground	UTCF2901N5	Basidiospores	LC804905
D16	Plantation	*C. japonica*	2022	Contact with the ground	UTCF2901N6	Basidiospores	LC804906
D17	Plantation	*C. japonica*	2022	Contact with the ground	UTCF2901N7	Basidiospores	LC804907
D18	Plantation	*C. japonica*	2022	Contact with the ground	UTCF2901N8	Basidiospores	LC804908
D19	Plantation	*C. japonica*	2023	Contact with the ground		Could not isolate	
D20	Plantation	*C. pisifera*	2021	Contact with the ground	UTCF3112J12	Basidiospores	LC804830

Results of PCR using species-specific primer set of *S. himantioides* were all positive.

Table S2. List of trees with *Serpula himantioides* fruiting bodies (continuation).

Tree number	Locality of source	Host	Collected Year	Bottom edge of fruiting body	Isolate number	Source of isolation	INSD accession number: ITS
D21	Plantation	*Chamaecyparis pisifera*	2021	Contact with the ground	UTCF3112J13	Basidiospores	LC804831
D22	Plantation	*C. pisifera*	2021	Contact with the ground	UTCF3112J14	Basidiospores	LC804832
D23	Plantation	*C. pisifera*	2021	Contact with the ground	UTCF3112J15	Basidiospores	LC804833
D24	Plantation	*C. pisifera*	2021	Contact with the ground	UTCF3112J16	Basidiospores	LC804834
D25	Plantation	*C. pisifera*	2021	Contact with the ground	UTCF3112J17	Basidiospores	LC804835
D26	Plantation	*Larix kaempferi*	2021	Contact with the ground	UTCF3114J18	Basidiospores	LC804836
D27	Plantation	*C. pisifera*	2021	Contact with the ground	UTCF3114J19	Basidiospores	LC804837
D28	Plantation	*C. pisifera*	2021	Contact with the ground	UTCF3114J20	Basidiospores	LC804838
D29	Plantation	*C. pisifera*	2021	Contact with the ground	UTCF3114J21	Basidiospores	LC804839
D30	Plantation	*C. pisifera*	2023	Contact with the ground	UTCF3112J34	Basidiospores	LC804843
D31	Plantation	*C. pisifera*	2023	Contact with the ground		Could not isolate	
D32	Plantation	*Abies homolepis* （Dead tree）	2023	Contact with the ground	UTCF2901N10	Basidiospores	LC816588
D33	Natural forest	Broadleaved tree (Dead tree）	2023	Contact with the ground	UTCF0329M11	Basidiospores	LC816589

Results of PCR using species-specific primer set of *S. himantioides* were all positive.

Table S3. List of *Serpula himantioides* fruiting bodies isolated from heartwood samples.

Locality of source	Host plant species	Isolate number	GenBank accession number: ITS
Plot A （Adjacent area）	*Chamaecyparis pisifera*	UTCF3112219	LC804909
Plot A （Adjacent area）	*C. pisifera*	UTCF3112498	LC804910
Plot B	*C. obtusa*	UTCF2805439L	LC804911
Plot B	*Larix kaempferi* (Dead tree)	UTCF2805T416	LC804912
Plot C	*C. obtuse*	UTCF3204333	LC804913
Plot C	*L. kaempferi* (Dead tree)	UTCF3204357	LC804914
Plantation	*Cryptomeria japonica*	UTCF2901S1	LC804915

Table S4. Density, composition, and mean diameter at breast height of living trees for each plot.

Survey year	2021			2022			2023		
Plot	A	B	C	A	B	C	A	B	C
Tree density of living trees (/ha)									
Chamaecyparis pisifera	1259	0	0	1245	0	0	1245	0	0
C. obtusa	0	622	722	0	608	722	0	608	722
*Larix kaempfer*i	0	283	311	0	255	297	0	241	297
Total	1259	905	1033	1245	863	1019	1245	849	1019
Composition of living trees (%)									
C. pisifera	100.0	0.0	0.0	100.0	0.0	0.0	100.0	0.0	0.0
C. obtusa	0.0	68.8	69.9	0.0	70.5	70.8	0.0	71.7	70.8
*L. kaempfer*i	0.0	31.2	30.1	0.0	29.5	29.2	0.0	28.3	29.2
Total	100.0	100.0	100.0	100.0	100.0	100.0	100.0	100.0	100.0
Mean diameter at breast height (cm)									
C. pisifera	31.9			32.5			32.9		
C. obtusa		20.5	20.0		20.8	19.8		20.8	20.0
*L. kaempfer*i		42.1	39.2		44.0	40.3		44.9	40.3
Total	31.9	27.2	25.8	32.5	27.7	25.9	32.9	27.7	25.9

Table S5. Number and incidence of dead trees with *Serpula himantioides* fruiting bodies.

**Survey year**	**2021**			**2022**			**2023**		
**Plot**	**A**	**B**	**C**	**A**	**B**	**C**	**A**	**B**	**C**
Number of dead trees									
Chamaecyparis pisifera	5	0	0	6	0	0	6	0	0
C. obtusa	0	1	5	0	2	5	0	2	5
*Larix kaempfer*i	0	3	4	0	5	5	0	6	5
Total	5	4	9	6	7	10	6	8	10
Number of dead trees with *Serpula himantioides* fruiting bodies									
C. pisifera	0	0	0	0	0	0	1	0	0
C. obtusa	0	0	1	0	0	1	0	0	1
*L. kaempfer*i	0	0	1	0	2	2	0	2	2
Total	0	0	2	0	2	3	1	2	3
Incidence of dead trees with *S. himantioides* fruiting bodies (%)									
C. pisifera	0.0			0.0			16.7		
C. obtusa		0.0	20.0		0.0	20.0		0.0	20.0
*L. kaempfer*i		0.0	25.0		40.0	40.0		33.3	40.0
Total	0.0	0.0	22.2	0.0	28.6	30.0	16.7	25.0	30.0

Table S6. List of *DFr* values using the lateral impact vibration method.

Tree Number	Plot	Tree Species	*DFr* Value		
			2021	2022	2023
A1	A	*Chamaecyparis pisifera*	**24.0**	**15.1**	**18.4**
A2	A	*C. pisifera*	29.3	30.1	**23.3**
A3	A	*C. pisifera*	**23.2**	**6.9**	**21.0**
A4	A	*C. pisifera*	**14.4**	**16.2**	15.7
A5	A	*C. pisifera*	**14.9**	**8.8**	**13.3**
A6	A	*C. pisifera*	**18.5**	**15.0**	**19.1**
A7	A	*C. pisifera*	26.3	30.8	25.6
A8	A	*C. pisifera*	**23.5**	**11.9**	**22.6**
A9	A	*C. pisifera*	**27.1**	**15.1**	**23.5**
A10	A	*C. pisifera*	**20.8**	**13.5**	**18.7**
A11	A	*C. pisifera*	**22.7**	**12.6**	**21.6**
A12	A	*C. pisifera*	**13.4**	**17.5**	**15.0**
A13	A	*C. pisifera*	**16.0**	**9.3**	**13.1**
A14	A	*C. pisifera*	**14.3**	**12.8**	**9.8**
A15	A	*C. pisifera*	28.2	31.7	29.1
A16	A	*C. pisifera*	28.1	31.2	27.1
A17	A	*C. pisifera*	**19.3**	**19.3**	**18.0**
A18	A	*C. pisifera*	**12.0**	**9.8**	**6.9**
A19	A	*C. pisifera*	**17.6**	**7.9**	**20.8**
A20	A	*C. pisifera*	**16.6**	**19.9**	**14.5**
A21	A	*C. pisifera*	25.3	31.2	25.7
A22	A	*C. pisifera*	30.3	Dead	Dead
A23	A	*C. pisifera*	27.0	29.1	27.1
A24	A	*C. pisifera*	**22.9**	**18.5**	**18.1**
A25	A	*C. pisifera*	29.4	**22.7**	**21.7**
A26	A	*C. pisifera*	**21.5**	**23.8**	**15.0**
A27	A	*C. pisifera*	29.6	**23.8**	**22.6**
A28	A	*C. pisifera*	27.7	30.1	25.0
A29	A	*C. pisifera*	29.1	32.6	28.0
A30	A	*C. pisifera*	29.4	31.0	27.4
A31	A	*C. pisifera*	**24.3**	**24.3**	**24.5**
A32	A	*C. pisifera*	29.9	32.0	28.3
A33	A	*C. pisifera*	27.8	26.3	**24.2**
A34	A	*C. pisifera*	**17.5**	**19.7**	**18.8**
A35	A	*C. pisifera*	30.7	30.8	30.1
A36	A	*C. pisifera*	28.5	30.1	28.0
A37	A	*C. pisifera*	28.7	31.8	29.0
A38	A	*C. pisifera*	26.8	30.2	27.3
A39	A	*C. pisifera*	25.1	25.1	**19.3**
A40	A	*C. pisifera*	**16.5**	**8.3**	**6.2**
A41	A	*C. pisifera*	27.4	33.1	29.8

Note: Values in bold indicate the presence of internal decay, N.A. indicate not subject to measurement. The presence of internal decay for *C. pisifera*, *C. obtusa*, and *L. kaempferi* was determined when the *DFr* value was less than 24.8 as *C. pisifera*, 27.1 as *C. obtusa*, and 24.2 as *L. kaempferi*.

Table S6. List of *DFr* values using the lateral impact vibration method (continuation).

Tree Number	Plot	Tree Species	*DFr* Value 2021	2022	2023
A42	A	*Chamaecyparis pisifera*	29.6	**23.2**	**20.5**
A43	A	*C. pisifera*	30.8	33.5	31.3
A44	A	*C. pisifera*	25.6	**16.8**	**16.3**
A45	A	*C. pisifera*	**24.1**	**24.1**	**23.3**
A46	A	*C. pisifera*	28.5	30.2	27.4
A47	A	*C. pisifera*	**23.5**	**13.4**	**24.1**
A48	A	*C. pisifera*	31.7	33.9	31.9
A49	A	*C. pisifera*	**21.2**	**12.2**	**10.1**
A50	A	*C. pisifera*	**12.8**	**10.6**	**10.9**
A51	A	*C. pisifera*	30.1	31.3	26.8
A52	A	*C. pisifera*	**14.3**	**16.7**	**10.0**
A53	A	*C. pisifera*	**15.0**	**19.6**	**18.8**
A54	A	*C. pisifera*	27.4	**20.4**	**10.6**
A55	A	*C. pisifera*	**24.1**	**13.5**	**9.0**
A56	A	*C. pisifera*	29.6	32.7	29.8
A57	A	*C. pisifera*	30.2	33.1	30.7
A58	A	*C. pisifera*	30.1	33.7	31.3
A59	A	*C. pisifera*	**17.7**	**8.5**	**15.4**
A60	A	*C. pisifera*	29.7	32.2	29.1
A61	A	*C. pisifera*	30.7	34.3	33.1
A62	A	*C. pisifera*	**18.7**	**18.7**	**22.0**
A63	A	*C. pisifera*	32.8	32.8	26.4
A64	A	*C. pisifera*	26.3	29.4	25.6
A65	A	*C. pisifera*	**22.5**	**22.5**	**20.7**
A66	A	*C. pisifera*	**11.7**	**15.0**	**16.9**
A67	A	*C. pisifera*	**19.3**	**18.5**	**21.1**
A68	A	*C. pisifera*	**18.9**	**20.3**	**17.8**
A69	A	*C. pisifera*	**22.7**	**19.0**	**13.9**
A70	A	*C. pisifera*	27.8	33.4	28.6
A71	A	*C. pisifera*	30.2	**23.2**	**21.5**
A72	A	*C. pisifera*	**11.7**	**9.5**	**8.3**
A73	A	*C. pisifera*	28.6	34.6	27.9
A74	A	*C. pisifera*	**12.9**	**11.6**	**9.7**
A75	A	*C. pisifera*	19.3	20.3	21.0
A76	A	*C. pisifera*	12.9	7.4	12.3
A77	A	*C. pisifera*	29.3	33.6	29.4
A78	A	*C. pisifera*	**18.0**	**9.3**	**18.6**
A79	A	*C. pisifera*	**23.4**	**21.3**	**18.2**
A80	A	*C. pisifera*	**22.7**	**11.3**	**19.1**
A81	A	*C. pisifera*	28.7	31.8	29.4
A82	A	*C. pisifera*	**14.3**	**6.9**	**21.3**

Note: Values in bold indicate the presence of internal decay, N.A. indicate not subject to measurement. The presence of internal decay for *C. pisifera*, *C. obtusa*, and *L. kaempferi* was determined when the *DFr* value was less than 24.8 as *C. pisifera*, 27.1 as *C. obtusa*, and 24.2 as *L. kaempferi*.

Table S6. List of *DFr* values using the lateral impact vibration method (continuation).

Tree Number	Plot	Tree Species	*DFr* Value 2021	2022	2023
A83	A	*Chamaecyparis pisifera*	**12.9**	**17.3**	**13.5**
A84	A	*C. pisifera*	28.5	34.1	26.2
A85	A	*C. pisifera*	30.7	33.7	31.1
A86	A	*C. pisifera*	**18.8**	**10.0**	**7.7**
A87	A	*C. pisifera*	**20.8**	**15.1**	**16.7**
A88	A	*C. pisifera*	**15.1**	**20.8**	**21.2**
B1	B	*C. obtusa*	29.4	32.8	32.8
B2	B	*Larix kaempferi*	31.8	30.9	30.9
B3	B	*C. obtusa*	34.7	34.8	33.7
B4	B	*C. obtusa*	33.7	33.6	33
B5	B	*C. obtusa*	32.7	33.7	34.6
B6	B	*C. obtusa*	7.6	7.6	7.6
B7	B	*C. obtusa*	30.1	29.6	29.6
B8	B	*C. obtusa*	N.A.	29.3	29.3
B9	B	*C. obtusa*	**14.8**	**14.8**	**14.8**
B10	B	*L. kaempferi*	27.7	28.9	28
B12	B	*L. kaempferi*	29.4	28.4	28.4
B15	B	*L. kaempferi*	27.1	27.0	Dead
B16	B	*L. kaempferi*	25.6	25.7	27.6
B17	B	*C. obtusa*	30.7	30.6	30.6
B18	B	*L. kaempferi*	28.5	28.5	28.5
B19	B	*L. kaempferi*	32.5	32.6	32.6
B20	B	*L. kaempferi*	32.9	Dead	Dead
B22	B	*L. kaempferi*	**23.6**	**23.7**	**23.7**
B23	B	*L. kaempferi*	27.4	29.1	28
B24	B	*L. kaempferi*	25.5	25.9	29.5
B25	B	*L. kaempferi*	27.7	27.7	26.8
B26	B	*C. obtusa*	35.1	35.1	35.1
B27	B	*L. kaempferi*	30.9	30.3	29.3
B28	B	*C. obtusa*	32.2	33.1	35.1
B29	B	*L. kaempferi*	27.3	28	28
B30	B	*C. obtusa*	33.9	34.7	34.1
B31	B	*C. obtusa*	31	31.3	31.9
B32	B	*C. obtusa*	33.4	33	33.5
B33	B	*C. obtusa*	30.8	33	32.5
B34	B	*L. kaempferi*	26.8	27.8	27.8
B35	B	*L. kaempferi*	32.4	30.9	30.1
B36	B	*C. obtusa*	30.7	30.7	30.7
B37	B	*C. obtusa*	34.3	34.3	34.3
B38	B	*L. kaempferi*	27.1	27.2	27.2
B39	B	*C. obtusa*	N.A.	N.A.	29.7

Note: Values in bold indicate the presence of internal decay, N.A. indicate not subject to measurement. The presence of internal decay for *C. pisifera*, *C. obtusa*, and *L. kaempferi* was determined when the *DFr* value was less than 24.8 as *C. pisifera*, 27.1 as *C. obtusa*, and 24.2 as *L. kaempferi*.

Table S6. List of *DFr* values using the lateral impact vibration method (continuation).

Tree Number	Plot	Tree Species	***DFr* Value**		
			2021	2022	2023
B40	B	*Chamaecyparis obtusa*	28.0	29.6	31.3
B41	B	*C. obtusa*	30.3	30.3	30.3
B42	B	*C. obtusa*	*12.9*	*12.9*	*12.9*
B43	B	*C. obtusa*	*23.7*	*22.7*	*22.7*
B44	B	*Larix kaempferi*	26.1	26.7	**22.8**
B45	B	*C. obtusa*	31.3	31.3	32
B46	B	*C. obtusa*	31.9	33.2	31.3
B47	B	*L. kaempferi*	**23.6**	Dead	Dead
B48	B	*C. obtusa*	32.7	33.1	33.1
B49	B	*C. obtusa*	39.2	28.1	37.7
B50	B	*L. kaempferi*	29.4	29.4	29.4
B51	B	*C. obtusa*	36.0	30.7	30.7
C1	C	*C. obtusa*	33.1	27.8	29.4
C2	C	*L. kaempferi*	22.6	27.5	26.8
C3	C	*C. obtusa*	28.8	28.1	28.1
C4	C	*L. kaempferi*	28.3	25.7	25.7
C5	C	*C. obtusa*	N.A.	33.7	34.1
C9	C	*C. obtusa*	30.8	**27.1**	**27.1**
C10	C	*L. kaempferi*	28.0	28.0	28.8
C11	C	*L. kaempferi*	29.4	28.6	28.6
C12	C	*L. kaempferi*	31.6	30.1	30.1
C13	C	*C. obtusa*	31.1	31.1	30.6
C14	C	*C. obtusa*	32.5	31.3	31.3
C16	C	*C. obtusa*	32.9	32.9	31.6
C17	C	*C. obtusa*	36.4	32.6	33.1
C18	C	*L. kaempferi*	29.9	28.0	28.0
C19	C	*C. obtusa*	N.A.	N.A.	35.1
C21	C	*L. kaempferi*	28.7	26.8	**23.0**
C22	C	*C. obtusa*	29.6	**27.1**	**27.1**
C23	C	*L. kaempferi*	**17.8**	Dead	Dead
C24	C	*L. kaempferi*	30.9	29.2	29.9
C28	C	*L. kaempferi*	30.5	30.3	30.3
C30	C	*C. obtusa*	33.0	28.1	30.3
C31	C	*C. obtusa*	N.A.	30.6	31.6
C32	C	*C. obtusa*	32.5	32.5	32.5
C33	C	*L. kaempferi*	32.9	30.4	30.4
C34	C	*C. obtusa*	31.2	29.4	28.2
C35	C	*L. kaempferi*	27.7	25.7	25.7
C36	C	*C. obtusa*	**14.0**	**14.0**	**14.0**
C37	C	*L. kaempferi*	31.4	29.9	29.4
C39	C	*C. obtusa*	33.4	31.5	31.5

Note: Values in bold indicate the presence of internal decay, N.A. indicate not subject to measurement. The presence of internal decay for *C. pisifera*, *C. obtusa*, and *L. kaempferi* was determined when the *DFr* value was less than 24.8 as *C. pisifera*, 27.1 as *C. obtusa*, and 24.2 as *L. kaempferi*.

Table S6. List of *DFr* values using the lateral impact vibration method (continuation).

Tree Number	Plot	Tree Species	*DFr* Value		
			2021	2022	2023
C40	C	*Larix kaempferi*	34.0	31.9	31.4
C41	C	*L. kaempferi*	29.8	27.8	27.8
C42	C	*L. kaempferi*	32.9	25.9	25.9
C43	C	*Chamaecyparis obtusa*	32.8	31.1	29.9
C44	C	*C. obtusa*	33.2	29.8	29.8
C45	C	*C. obtusa*	30.5	27.6	**27.1**
C46	C	*L. kaempferi*	29.3	29.2	29.2
C47	C	*C. obtusa*	33.6	32.0	31.4
C48	C	*C. obtusa*	N.A.	28.9	28.9
C49	C	*L. kaempferi*	32.6	31.4	30.2
C50	C	*C. obtusa*	30.8	28.4	27.7
C51	C	*C. obtusa*	31.4	27.6	27.6
C52	C	*L. kaempferi*	28.7	27.0	27.0
C53	C	*C. obtusa*	29.0	**25.2**	**25.2**
C54	C	*C. obtusa*	29.6	**26.5**	**26.5**
C55	C	*C. obtusa*	N.A.	N.A.	30.2
C56	C	*C. obtusa*	31.8	28.5	28.5
C57	C	*L. kaempferi*	29.5	27.6	27.6
C58	C	*C. obtusa*	29.3	**26.7**	**26.0**
C59	C	*C. obtusa*	N.A.	N.A.	31.8
C60	C	*L. kaempferi*	30.9	29.9	29.9
C61	C	*C. obtusa*	28.9	28.9	28.9
C62	C	*L. kaempferi*	27.3	**24.2**	**20.0**
C63	C	*C. obtusa*	31.6	28.2	28.2

Note: Values in bold indicate internal the presence of decay, N.A. indicate not subject to measurement. The presence of internal decay for *C. pisifera*, *C. obtusa*, and *L. kaempferi* was determined when the *DFr* value was less than 24.8 as *C. pisifera*, 27.1 as *C. obtusa*, and 24.2 as *L. kaempferi*.

Table S7. Number of living trees with *Serpula himantioides* fruiting bodies by single-year and multi-year occurrence.

Plot	Single year occurrence	Multi-year occurrence
A	10	11
B	6	15
C	7	25

Note: Trees that were live as of 2023.

Table S8. Number, mean diameter at breast height, and mean height of fruiting bodies of living trees by species used for GLM and GLMM.

Tree species	Survey Year	Fruiting bodies	Number of living trees[1]	Mean *DFr*	Mean DBH (cm)	Mean height of fruiting body (cm)
Chamaecyparis pisifera	2021	Development	11	22.2	36.4	33.0
		Not development	77	23.5	31.5	0.0
		Total	88	23.3	32.1	4.6
	2022	Development	14	16.8	38.1	28.6
		Not development	73	22.9	31.6	0.0
		Total	87	21.9	32.7	4.6
	2023	Development	8	19.2	37.1	16.6
		Not development	79	21.3	32.7	0.0
		Total	87	21.1	33.1	1.5
C. obtusa	2021	Development	18	29.8	37.1	79.2
		Not development	32	30.5	32.7	0.0
		Total	50	30.3	33.1	28.5
	2022	Development	22	29.1	24.6	83.8
		Not development	32	29.1	24.6	0.0
		Total	54	29.1	24.6	34.1
	2023	Development	22	28.6	24.3	49.6
		Not development	36	30.0	24.0	0.0
		Total	58	29.5	24.1	18.8
*Larix kaempfer*i	2021	Development	14	27.5	37.0	51.3
		Not development	28	29.4	42.4	0.0
		Total	42	28.8	40.6	17.1
	2022	Development	15	28.5	40.4	57.5
		Not development	24	28.2	43.0	0.0
		Total	39	28.3	42.0	22.1
	2023	Development	16	28.1	42.8	42.9
		Not development	22	28.0	42.0	0.0
		Total	38	28.0	42.3	18.1

1: Excluding living trees that were not subject to decay diagnosis.

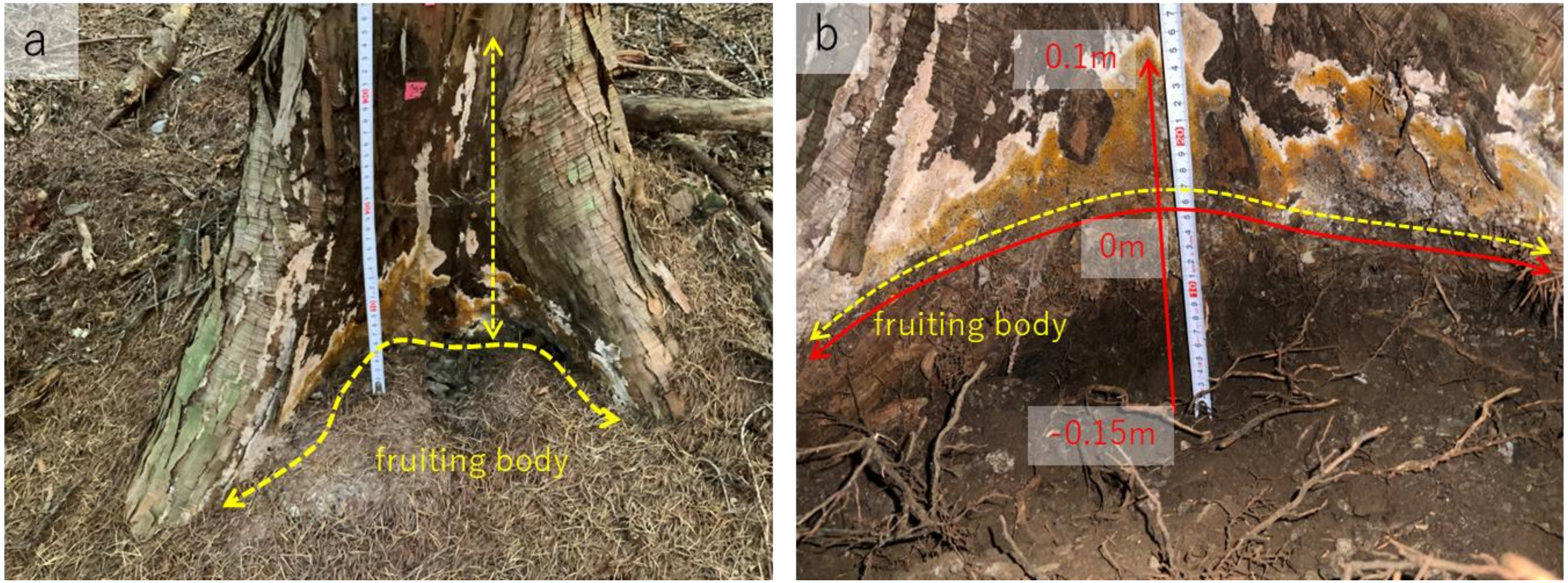

Figure S1. Example of underground condition of living trees with *Serpula himantioides* fruiting bodies. (a) *Chamaecyparis obtusa* with *S. himantioides* fruiting body (B4 in Table S2); (b) Example with removed soil. Fruiting bodies were not observed underground. Red numbers indicate the height above ground level, while the yellow line indicates the extent of fruiting body development.

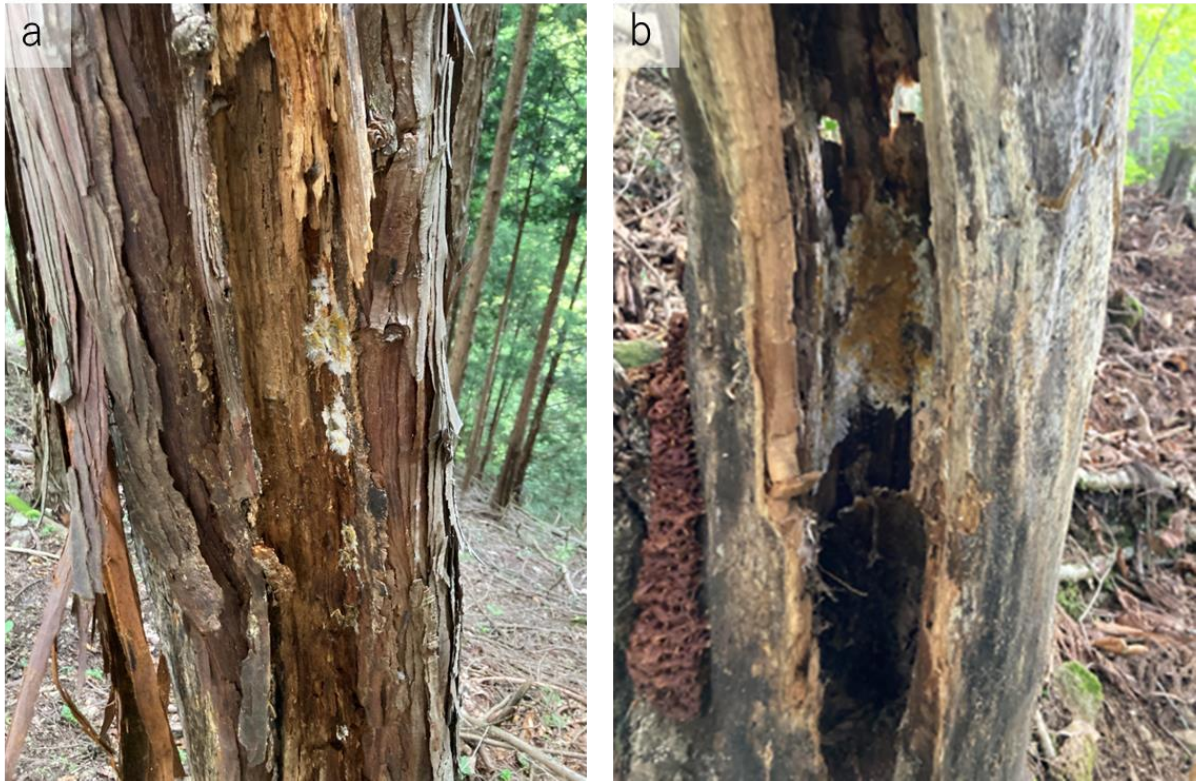

Figure S2. Examples of S*erpula himantioides* fruiting bodies that do not have a bottom edge touching the ground. (a) Fruiting body on the trunk of *Chamaecyparis obtusa*; (b) Fruiting body of a dead broadleaved tree (unknown species).

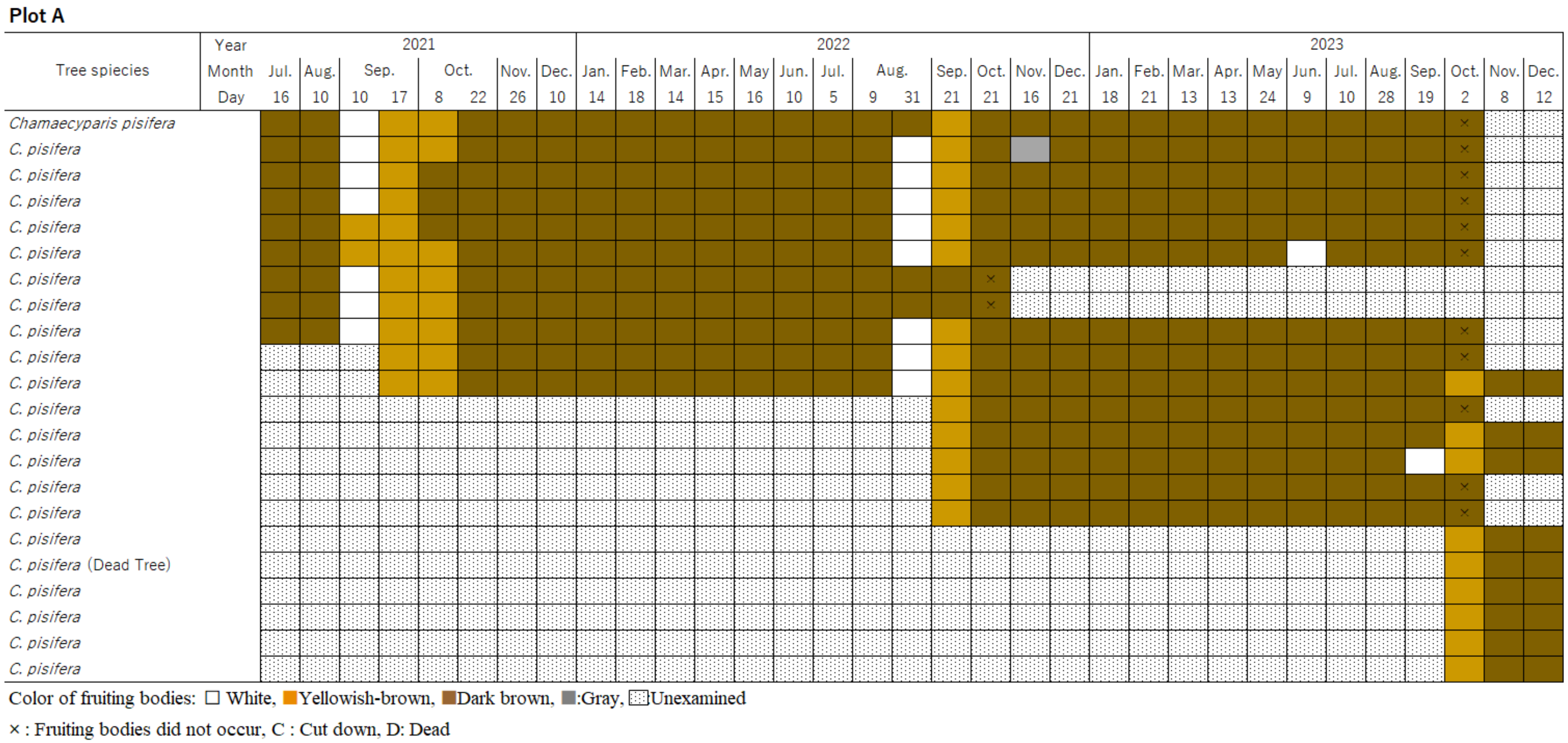

Figure S3. Color change of *Serpula himantioides* fruiting bodies.

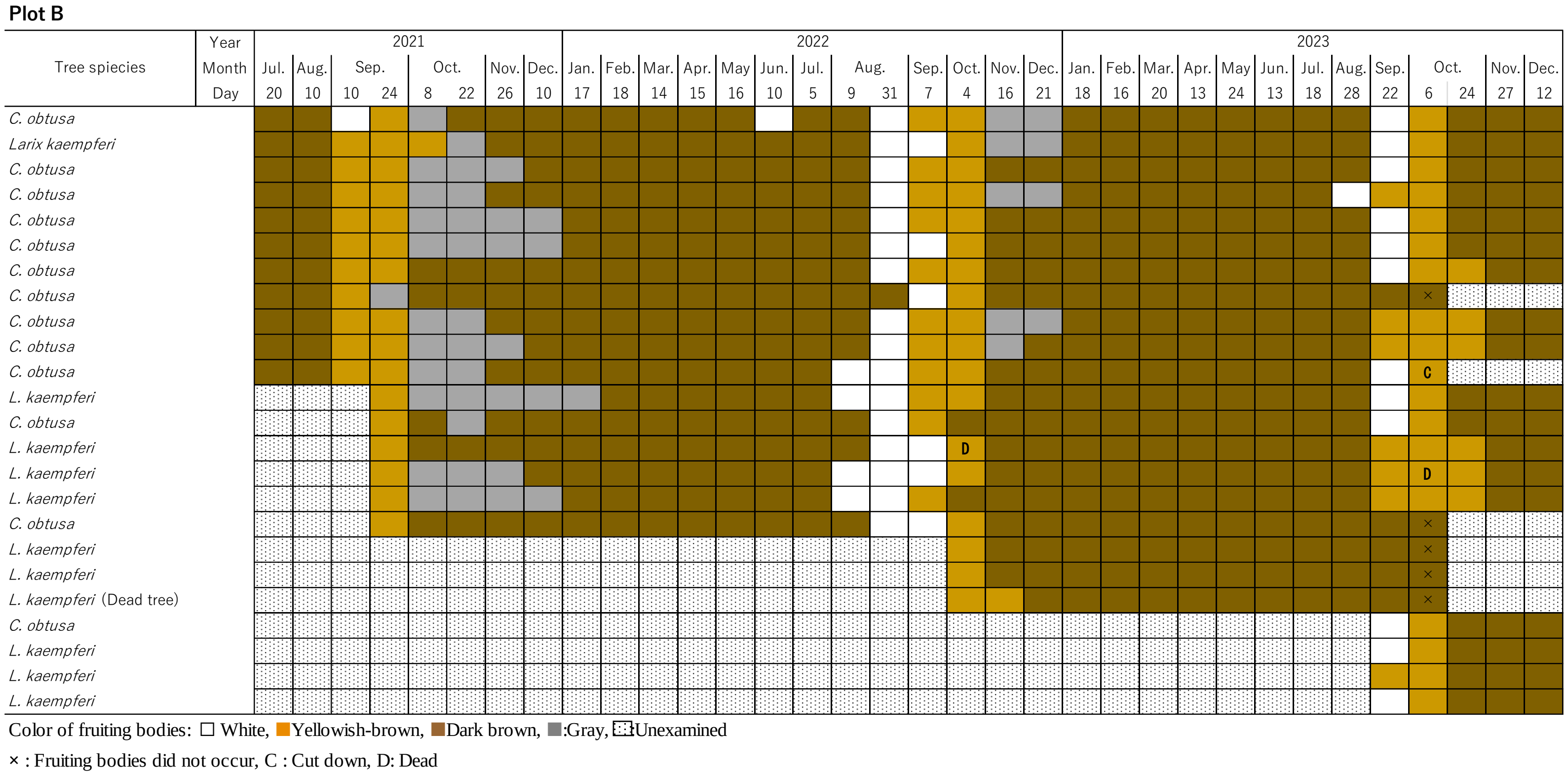

Figure S3. Color change of *Serpula himantioides* fruiting bodies (continuation).

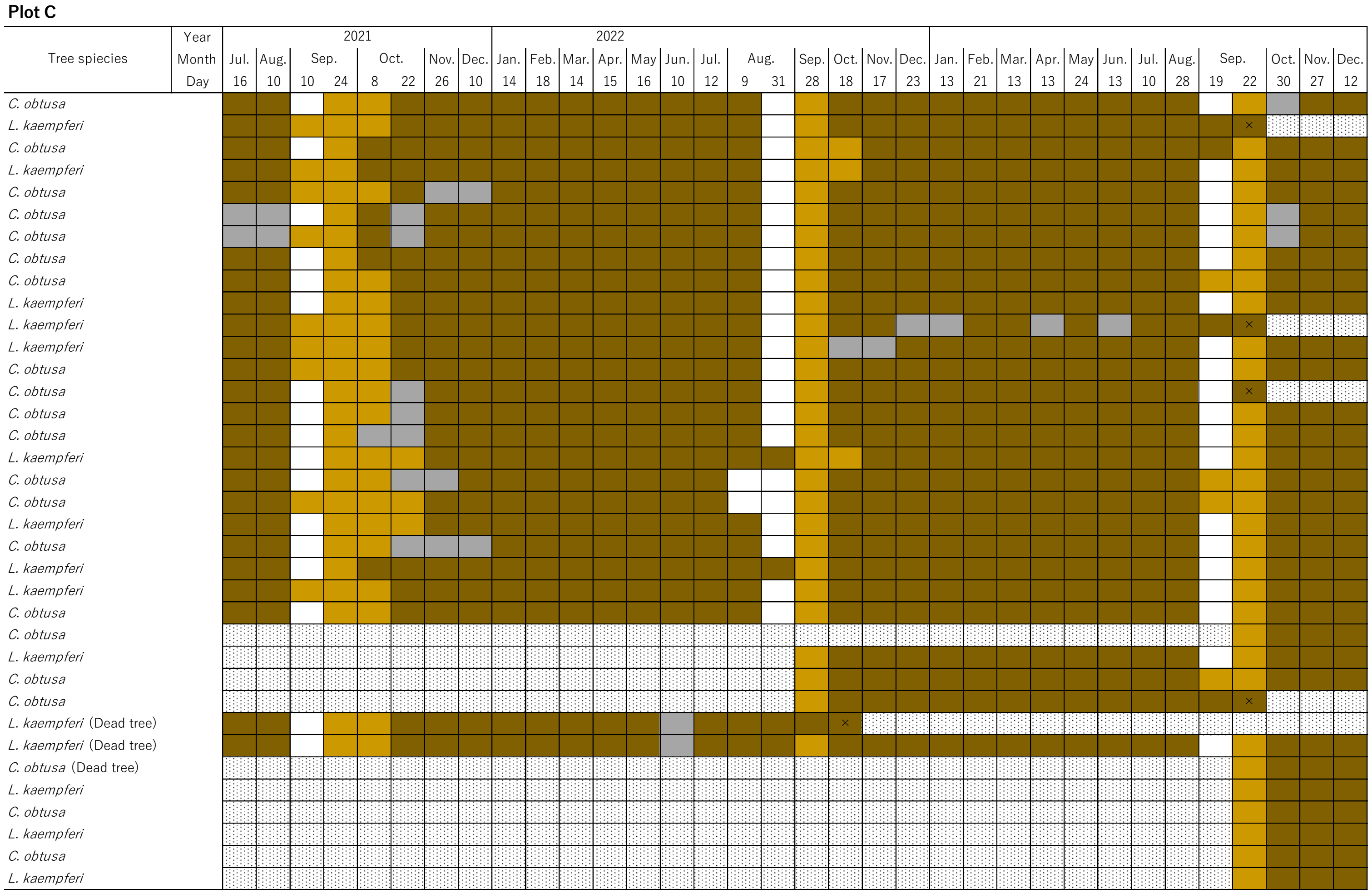

Color of fruiting bodies: ☐ White, ■Yellowish-brown, ■Dark brown, ■:Gray, ⬚Unexamined

× : Fruiting bodies did not occur, C : Cut down, D: Dead

Figure S3. Color change of *Serpula himantioides* fruiting bodies (continuation).

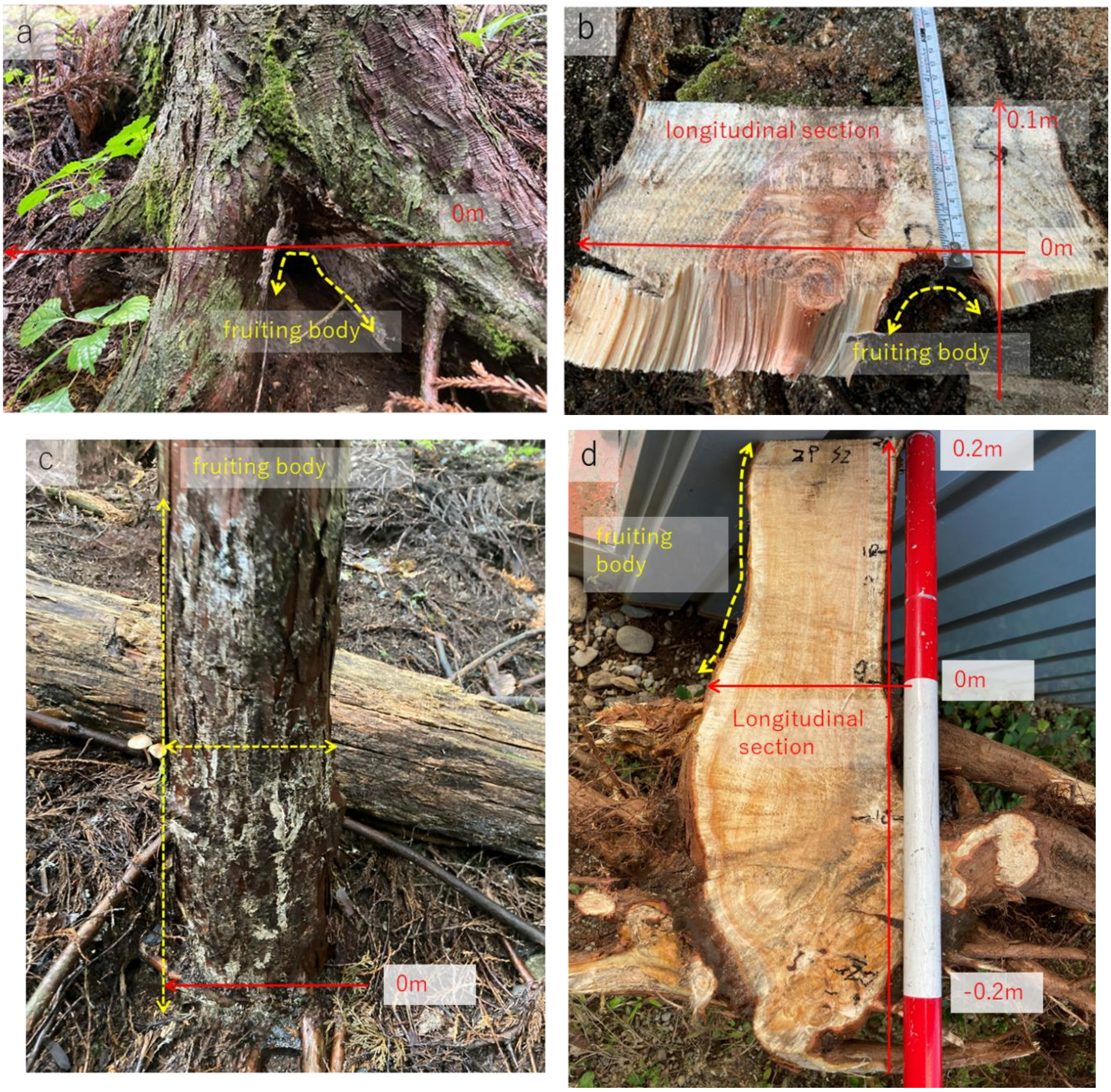

Figure S4. Dismantled living trees with *Serpula himantioides* fruiting bodies. (a) *Cryptomeria japonica* with *S. himantioides* fruiting body; (b) Longitudinal section of (a). The fruiting body did not invade the heartwood and no discoloration of the heartwood was observed. (c) *Chamaecyparis obtusa* with *Serpula himantioides* fruiting body; (d) Longitudinal section of (c). The color of the fruiting bodies was dark brown because they were cut in November. The fruiting body did not invade the heartwood, and no heartwood decay was observed. Red numbers indicate the height above ground level and the yellow line indicates the extent of fruiting body development.

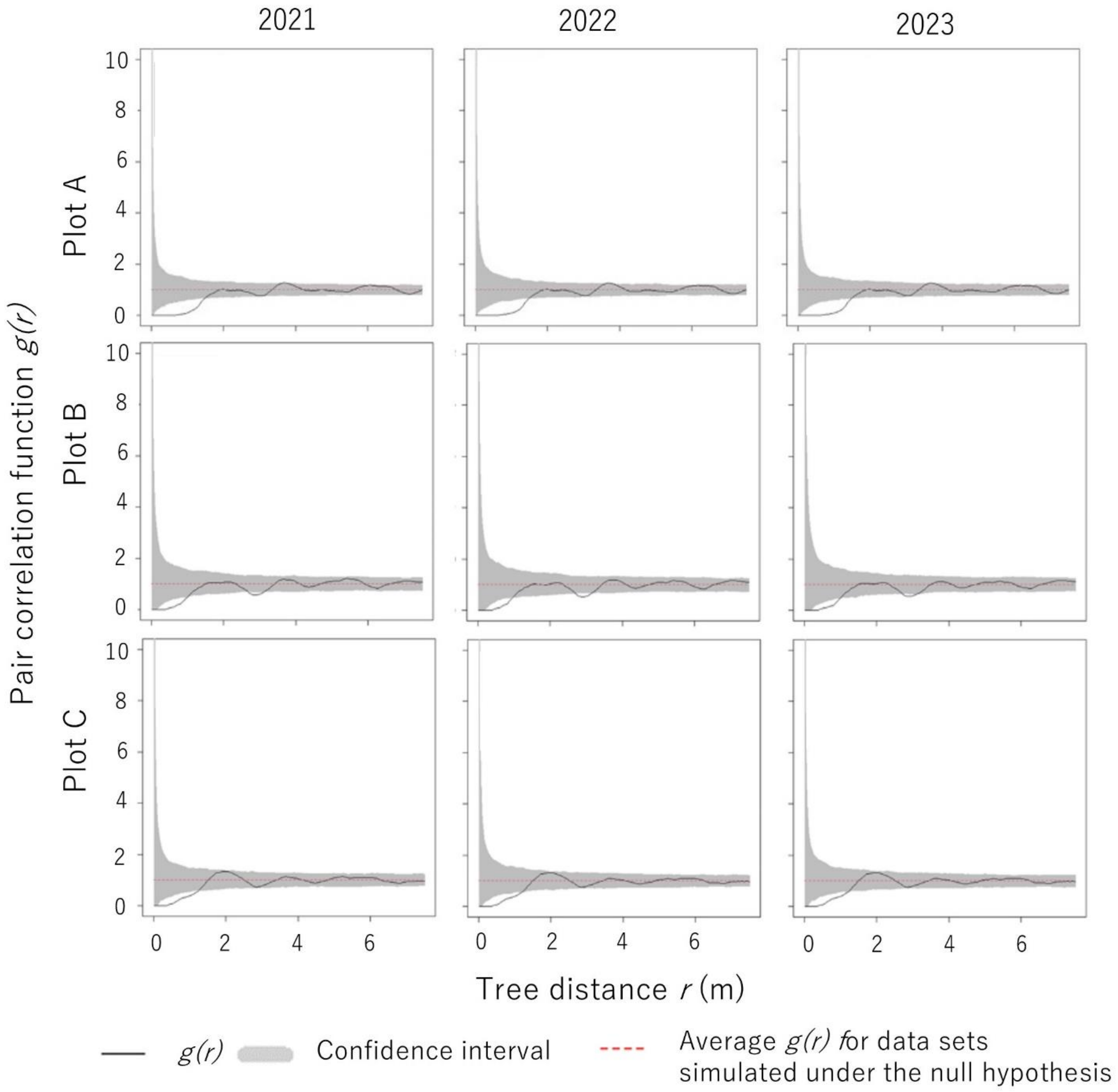

Figure S5. Pair correlation function showing the spatial distribution pattern of all living trees. Solid lines show the actual spatial distribution pattern in the plot, gray lines show the 95% confidence interval after 999 Monte Carlo simulations, and dashed lines show the mean value from the simulations. *g(r)* values greater than the confidence interval indicate an aggregated population distribution; *g(r)* values lower than the confidence interval, a uniformly distributed population; and *g(r)* values within the confidence interval, a randomly distributed population.
